# Preparation of stable colloidal dispersion of surface modified Fe_3_O_4_ nanoparticles for magnetic heating applications

**DOI:** 10.1038/s41598-024-51801-5

**Published:** 2024-01-14

**Authors:** Behnam Sabzi Dizajyekan, Arezou Jafari, Mohsen Vafaie-Sefti, Reza Saber, Zahra Fakhroueian

**Affiliations:** 1 Chemical Engineering Faculty, Tarbiat Modares University, Tehran, Iran; 2 Advanced Medical Technologies and Equipment Institute, Tehran University of Medical Sciences (TUMS), Tehran, Iran; 3 School of Chemical Engineering, College of Engineering, IPE, University of Tehran, P. O. Box 11155‑4563, Tehran, Iran

**Keywords:** Nanoparticle synthesis, Targeted therapies, Drug delivery, Nanocomposites

## Abstract

The effect of surface modification on enhancing the magnetic heating behavior of magnetic nano fluids were investigated, for this purpose Fe_3_O_4_ nanoparticles were synthesized using co-precipitation method and surface modification was done using citric acid, ascorbic acid, tetraethyl orthosilicate (TEOS), polyvinyl alcohol (PVA) and polyethylene glycol (PEG). Experimental heating tests using AC magnetic field were done in the frequency of 100 kHz and different magnetic field (H) intensities. Theoretically the specific absorption rate (SAR) in magnetic nano fluids is independent of nanoparticles concentration but the experimental results showed different behavior. The theoretical SAR value @ H = 12kA.m^–1^ for Nano fluids containing bare Fe_3_O_4_ nanoparticles was 11.5 W/g but in experimental tests the obtained value was 9.72 W/g for nano fluid containing 20,000 ppm of dispersed nanoparticles. The experimental SAR calculation was repeated for sample containing 10,000 ppm of nanoparticles and the results showed increase in experimental SAR that is an evidence of nanoparticles agglomeration in higher concentrations. The surface modification has improved the dispersion ability of the nanoparticles. The Ratio of SAR_, experimental, 20000ppm_ to SAR_, experimental, 10000ppm_ was 0.85 for bare Fe_3_O_4_ nanoparticles dispersion but in case of surface modified nanoparticles this ratio has increased up to 0.98 that shows lower agglomeration of nanoparticles as a result of surface modification, although on the other hand the surface modification agents were magnetically passive and so it is expected that in constant concentration the SAR for bare Fe_3_O_4_ nanoparticles to be higher than this variable for surface modified nanoparticles. At lower concentrations the dispersions containing bare Fe_3_O_4_ nanoparticles showed higher SAR values but at higher concentrations the surface modified Fe_3_O_4_ nanoparticles showed better results although the active agent amount was lower at them. Finally, it should be noted that the nanoparticles that were surface modified using polymeric agents showed the highest decrease in experimental SAR amounts comparing theoretical results that was because of the large molecules of polymers comparing other implemented surface modification agents.

## Introduction

Fe_3_O_4_ superparamagnetic nanoparticles (SPMNP) have significant potential applications in various science branches including drug delivery^1^ rheological enhancement of fluids^2^, heat generation in magnetic field^3,4^ and contrast enhancement in magnetic resonance imaging^5^. Magnetic nanoparticles (MNP) may be modulated by external magnetic fields to penetrate directly into the tumor, making them suitable drug carriers in medical applications and specifically drugs delivery. It has been demonstrated that the sizes, shapes, and surface coatings of MNPs have an important impact in the delivery of drugs in tumor treatment. MNPs having a high surface-to-volume ratio and porosity are favorable for drug delivery systems^6^. Magnetorheological behaviors in fluids are caused by dispersing magnetic nanoparticles (transition metal magnetic oxides) with polymer covering to guarantee stable dispersion in fluids. The formation of a magnetic field in magnetorheological fluids results in the formation of a chain structure of nanoparticles in a relatively short period, causing the viscosity of the fluid to alter. When the magnetic field is removed, these chains vanish, causing the rheological behavior to revert to its original condition^7^. Recently, many efforts have been made to explore the heating of magnetic nanoparticle-containing dispersions in an alternating magnetic field as well as the prevailing process and it has shown that relaxation mechanisms generate more heat than hysteresis mechanisms^8^. Furthermore, due to their biocompatibility, superior magnetic characteristics, and ability to functionalize their surfaces with diverse ligands for molecular MRI, iron oxide nanoparticles are being widely researched as contrast agents for magnetic resonance imaging (MRI)^9^.

One of the suitable methods for synthesis of these nanoparticles is co-precipitation because in this method the effective parameters can be controlled more easily and the synthesis conditions are not severe^10^. Number of parameters must be controlled in co-precipitation method to obtain repeatable results, including Fe^3+^ to Fe^2+^ ratio, reaction temperature, final pH, Fe salts type (sulfate or chloride etc., …), base type (NH_4_OH or NaOH etc.,…), mixing rate, sequence of addition of reactants and using or not using inert gas for oxidation prevention during synthesis^11–19^.

Surface modification of the nanoparticles of transient metals oxides (especially Fe) is essential to distribute them evenly in target solution. Moreover, in medical applications of magnetite nanoparticles, surface modification of Fe_3_O_4_ nanoparticles is crucial in making them biocompatible^20^, so different agents such as polymeric materials, inorganic materials and surfactants have been used in the researches for surface modification of these nanoparticles^21–24^. Several studies have recently reported on the modification of the surface of magnetite nanoparticles and improvement of their properties for various applications, including: collection of spilled oils on the earth’s surface^25^, removal of coomassie brilliant blue-R250 dye (CBB) from aqueous solutions^26^, dye absorption^27^, inhibiting hen egg-white lysozyme (HEWL) fibrillization and destroying mature fibrils^28^. Polyamines^25^, multifunctional pyridinium ionic liquids^26^, citric acid^28^, trisodium citrate^28^, dopamine conjugates^29^, oleic acid and polyacrylic acid^30^ were utilized in these studies to modify the surface of nanoparticles. Using different agents to modify the surface of nanoparticles yields unique properties, such as the ability to reuse magnetite nanoparticles modified with polyamines^25^, increasing the absorption of coomassie brilliant blue-R250 dye (CBB) from aqueous solutions from 84.4 mg/g in case of nanoparticles without surface modification to more than 700 mg/g in case of surface modified magnetite nanoparticles^26^, The increase in dye absorption from 60% for nanoparticles without surface modification to 98% for those modified with citric acid and the effect on their anti-amyloid potential^27^, improving bioreactivity with dopamine conjugates^29^, and the increase in dispersibility in aqueous environments by surface modification using polyacrylic acid^30^, the significantly increased stability in saline water and the creation of hydrophobic properties with surface modification using oleic acid^31^ are all examples of these outcomes.

As mentioned earlier heat production in magnetic field is one of the applications of superparamagnetic nanoparticles. The produced heat can be used for beginning desired chemical reactions or enhancing physical features such as viscosity in the carrier fluid. The heat production by applying magnetic field on superparamagnetic nanoparticles has been studied in many researches^32–40^. From the standpoint of the high efficacy of hyperthermia, it is crucial to accurately estimate the quantity of heat produced. It is also vital to minimize the usage of animals in laboratory stages. By dispersing superparamagnetic particles in an environment of glycerol and agarose gel, which has characteristics similar to those of body tissues, and measuring the temperature with optical fiber thermometers, the pseudo-tumor environment system (P-TES) method has been able to produce results with an acceptable level of accuracy^41^. It has been reported that the synthesis of desirable materials, such as nano catalysts, can be aided by the quick and simultaneous selective heating by the magnetic field. Comparing magnetic heating to other heating techniques has revealed that magnetic heating performs significantly better in several laboratory experiments. This is as magnetic heating causes the material’s surface temperature to rise more quickly^42^. Materials suitable for use in heating with the help of magnetic field are not limited to one material and different chemical compounds have also been taken into consideration in different researches according to the environment used. For instance, in different environments, the amorphous composition of FeZrB has demonstrated a faster magnetic heating rate compared to Fe_3_O_4_, which has resulted in a substantial decrease in the time needed for the ambient temperature to reach a degree that is acceptable for hyperthermia. SAR in ferrite nanoparticles is around 27.2 W/g, while SAR in FeZrB nanoparticles is approximately 65 W/g^43^. Due to their stability, adequate heat absorption, good conduction coefficient, and magnetic heating, nanofluids containing Fe_3_O_4_.H_2_O are appropriate for application in solar collectors, heat exchangers, and automobile radiators^44^.

The reported specific absorption rate (SAR) values in magnetic heating tests varies in different researches for so many reasons. One of the most major reasons for the uncertainty in determining the SAR value is the lack of a proper setup for measuring the quantity of heat produced. The pulse-heating approach in adiabatic settings has provided pretty accurate SAR data, but its disadvantage is the necessity for an advanced setup^45^. In addition to effect of frequency and magnetic field strength that have been formulated in Rosensweig’s research^46^ the effect of nanofluid viscosity and size distribution of nanoparticles are important parameters that change the dominant mechanism of nanoparticles heat generation in AC magnetic field and so the reported results. It should be noted that the formula presented by Rosensweig is only valid for a range of frequency and intensity of the magnetic field in which linear response regime is established. Experimental research on the behavior of nanoparticles in greater field intensities and frequencies has shown that the quantity of heat produced is constant once it reaches the saturation level and does not change with increasing field intensity^47^. Determining the amount of heat released in the hyperthermia process is challenging and variable results have been mentioned in different studies. The two main methods for this work are calorimetry and AC magnetometry. It has been shown that if the frequency and intensity of the magnetic field are in the linear response regime, then the obtained results from these two methods are almost equal to each other^48^. Considering the temperature drop caused by the transfer of energy to the environment is also one of the measures used to reduce the error of SAR calculations in calorimetric methods. For this purpose, the corrected slope technique is employed^49^.

According to the formulas that have been presented in the literature the Neel relaxation time and Brownian relaxation time of a single nanoparticle are functions of nanoparticle size; therefore, in constant mean size but different size distribution of the nanoparticles, the dominant mechanism of heat generation in nanofluid may change from Neel to Brownian and so diverse SAR results has been reported in literature. Based on the formulations, increasing the viscosity of the nanofluid results in Brownian relaxation time increase and making the Neel mechanism as the dominant heating mechanism. In different scientific fields such as hyperthermia in body tissues, polymer processing or petroleum industry the carrier fluid is viscous and so the Brownian relaxation time to Neel relaxation time ratio is in the order of 10^3^ or more^3^.

It is noteworthy to mention that particles without a hysteresis loop generate heat only through Neel and Brownian relaxations, and in the event that one does, calculations pertaining to determining the area of the loop and the heat generated by it must also be accounted for. Additionally, even superparamagnetic particles exhibit a hysteresis loop at a temperature below the blocking temperature and the coercivity of these nanoparticles become zero above the blocking temperature^50^. Consequently, the theoretical computations of the generated heat should take into account the hysteresis loop calculations; however, it should also be noted that the measurements obtained during the vibrating sample magnetometry (VSM) testing indicate the static loop, but there is less chance of aggregation formation and less interaction between the particles when they are distributed in a fluid (in calorimetry tests) comparing to VSM test conditions and taking into account variations in the time constant, particle size, interactions^51,52^, ability to respond to an applied magnetic field^53^, and factors influencing the blocking temperature, it is not unlikely to alter the behavior of nanoparticles and change the size of the hysteresis loop when they are being distributed in a fluid^54^. The size of the hysteresis loop, and hence the heat produced by the hysteresis loops, is also affected by the applied waveform^55^.Taking these considerations into account, all feasible options should be included in magnetic heating theoretical calculations in order to produce the most accurate results.

Conducting the magnetic heating tests in a high viscous medium in order to eliminate the Brownian mechanism of heat generation and simulating the real applications condition is the aim of the current research. In order to do so the nanoparticles were evenly distributed in a viscous polymer solution to restrict the nanoparticles physical rotations in AC magnetic field and so increasing the ratio of Brownian relaxation time to Neel relaxation to more than 10^5^.

In this research Magnetic Fe_3_O_4_ nanoparticles were synthesized using co-precipitation method. The nanoparticles were dispersed in a polymer aqueous solution using a mechanical mixer. The viscosity of the polymer solution was high, so the effect of Neel relaxation mechanism on heating of the nanofluid containing Magnetic nanoparticles was investigated more preciously. Five different materials were used for surface modification of the magnetite nanoparticles and making them more dispersible in polymer solution. The used materials for surface modification were citric acid, ascorbic acid, Tetraethyl orthosilicate (TEOS), polyvinyl alcohol (PVA) and polyethylene glycol (PEG). Surface modified nanoparticles and bare Fe_3_O_4_ nanoparticles were dispersed in polymer solution. Fourier-transform infrared spectroscopy (FTIR) was used to characterize the functional groups on the nanoparticles. Also VSM was used to investigate the magnetic properties of the nanoparticles. Nanoparticles’ heating was done in five turn coil magnetic induction heating device with variable magnetic field strength and the effect of surface modification, magnetic field strength and nanoparticles’ concentration on the specific absorption rate (SAR) and final temperature of polymeric solution was investigated.

## Material and method

### Material

Ferric chloride hexahydrate (FeCl_3_.6H_2_O, Titrachem, Iran), ferrous chloride tetrahydrate, (FeCl_2_.4H_2_O, Titrachem, Iran), Ethanol (C_2_H_5_OH, Titrachem, Iran), Ammonia (NH_4_OH Titrachem, Iran), TEOS (Si(OC_2_H_5_)_4_, Titrachem, Iran) L-Ascorbic acid (C_6_H_8_O_6_, Loba chemie, India), Citric acid (C_6_H_8_O_7_, Loba chemie, India), Polyvinyl Alcohol (PVA) (Mw = 72,000 D) [CH_2_CH(OH)]_n_, Merck, Germany) and Polyethylene Glycol (PEG) (Mw = 6000 D) (H(OC_2_H_4_)_n_OH, Merck, Germany) without further purification were used in synthesis of surface modified nanoparticles. Co-polymer of 2-acrylamido-2-methylpropane sulfonic acid sodium salt (AMPS) and acrylamide (ACA), in powder form, under the trade name of AN125, with sulfonation degree of 25% and average molecular weight of 8 million Dalton were prepared from SNF Co. (Saint-Étienne, France) in order to prepare aqueous polymeric dispersion of nanoparticles.

### Synthesis of Fe_3_O_4_ nanoparticles

The synthesis of nanoparticles were done using the method that were used in our previous work with a little modification^56^. Briefly, a homogeneous solution of FeCl_3_.6H_2_O and FeCl_2_.4H_2_O with molar ratio of 2:1 was prepared in 250 ml of deionized water. The temperature was raised to 80 °C and N_2_ purging was done in order to eliminate the O_2_ gas in the solution and prohibit the unwanted oxidation of Fe_3_O_4_ to γ-Fe_2_O_3_^57^. 80 ml NH_4_OH solution (25 v/v %) was added to the solution drop wise in 60 min meanwhile the solution was stirring vigorously. In order to control the nanoparticles mean size and size distribution, the temperature and pH of the solution was being controlled during the synthesis continuously. Finally the reaction was allowed to be continued for another 60 min while the solution medium was being stirred vigorously and refluxed. The final pH of the solution was about 12. The nanoparticles were washed and decanted several times using deionized water and a 0.4T permanent magnet. The washing procedure was continued until the final decanted water became neutral using a PH meter, at this step the nanoparticles were maintained in degased (using N_2_) deionized water for further usage.

### Surface modification of the Fe_3_O_4_ nanoparticles

The surface modification of the nanoparticles was done similar to the method used in our previous work^56^ with a little modification. Briefly, the surface modification agent was added to the reaction medium right after the Fe_3_O_4_ synthesis reaction completion and without washing or drying the nanoparticles. The amount of surface modification agent was 40 wt% of stoichiometric produced Fe_3_O_4_. The surface modification was allowed to continue for 6 h.

The surface modification using TEOS was a little different. In this method ethanol and NH_4_OH was added to the produced Fe_3_O_4_ dispersion to enhance the TEOS hydrolysis reaction (Eq. 1). The stochiometric amount of TEOS was dissolved in 100 ml of ethanol and the solution was added drop wise while the reaction medium was being stirred vigorously and refluxed. TEOS hydrolyze through the Eq. 1 and produces SiO_2_ that cover the surface of Fe_3_O_4_ nanoparticles^23^.

1 $${\text{Si}}\left( {{\text{OC}}_{{2}} {\text{H}}_{{5}} } \right)_{{4}} + {\text{ 2H}}_{{2}} {\text{O }} \to {\text{ SiO}}_{{2}} + {\text{ 4C}}_{{2}} {\text{H}}_{{5}} {\text{OH}}{.}$$

**Figure 1:**
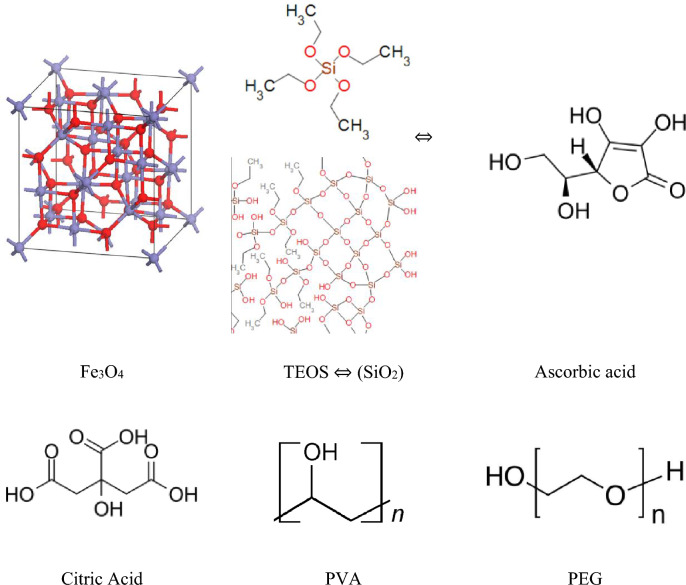
The molecular structure of the substances used in the research.

The molecular structure of the substances used in this research are shown in Fig. 1. The difference between electronegativity of oxygen and iron is 1.61 and the bonding is polar, so dipole–dipole interaction can be expected between polar molecules and Fe or O atoms in Fe_3_O_4_ crystal. The ascorbic acid and citric acid both have OH group which loose H^+^ in water and so makes them suitable to be attached to the surface of Fe_3_O_4_ crystal through Fe atoms. On the other hand, PVA has an OH group in each repeating group and so a strong enough interaction can be expected between PVA molecule and Fe_3_O_4_ nanoparticle’s crystal. In case of PEG there is only one OH group at one end of each PEG molecule so the interaction well be weak, also there is an ether group in each repeating unit but its’ not polar enough to expect a strong interaction between the PEG molecule and Fe_3_O_4_ crystal. Finally, in case of SiO_2_ it should be mentioned that SiO_2_ just coats the surface of Fe_3_O_4_ particles and it is not a surface modification agent.

### Characterization tests

*FTIR* (frontier spectrometer, PerkinElmer) was used to characterize the functional groups on the surface of the nanoparticles. The spectrum was taken in the range of 400 to 4000 cm^–1^.

*VSM* (vibrating sample magnetometer) (MDKB–Co, IRAN) was used for magnetic characterization of the synthesized magnetic nanoparticles and determining the saturation magnetization and magnetic susceptibility^58^ of the nanoparticles. The magnetization results was used to determine the magnetic core size (assuming log normal distribution) of the nanoparticles using the Eq. (2)^59,60^

2 $${D}_{m}=[\frac{18kT}{{\mu }_{0}\pi {M}_{d}} (\frac{{\chi }_{i}}{3\varepsilon {M}_{d}{H}_{0}}{)}^{1/2}{]}^{1/3}.$$

In the above equation D_m_ is the magnetic core diameter of the nanoparticles, ${\mu }_{0}$ is the permeability of the free space, M_d_ is the domain magnetization of the nanoparticles, ${\chi }_{i}$ is the initial susceptibility that will be determined using the slope of the M vs H diagram at H → 0 and finally H_0_ will be determined by plotting the M vs 1/H at high external field where the diagram become linear, the intercept on the M axis is the H_0_^61^.

### Theoretical determination of heat production in magnetic field

The energy produced by nanoparticles in a magnetic field is divided into three components: eddy current loss, hysteresis loss, and relaxation loss. For the theoretical measurement of the quantity of heat produced, many models such as the Rayleigh model, the Stoner-Wohlfarth model based theories (SWMBT), and the linear response model^62^ have been proposed based on the applicable conditions. To determine the application scope of each model, two dimensionless parameters $\kappa (=\frac{{k}_{B}T}{{K}_{eff}V}{\text{ln}}(\frac{{k}_{B}T}{4{\mu }_{0}H{M}_{s}Vf{\tau }_{0}}))$ and $\xi$(= $\frac{{\mu }_{0}{M}_{s}VH}{{k}_{B}T})$are used. In case ξ < 1, the entire heat generation may be assigned to the loss mechanism via relaxation^63^, and thus Rosensweig’s model^46^ can be utilized.

In trying to formulate the heat production in magnetic field Rosensweig^46^ presented the Eq. (3) for the amount of the produced heat in ferrofluids in AC magnetic field:

3 $$P= \pi {\mu }_{0}{\chi }_{0}{H}_{0}^{2}f\frac{2\pi f\tau }{1+(2\pi f\tau {)}^{2}}.$$

In the above equation ${\mu }_{0}$ is the permeability of free space ($4\pi \times {10}^{-7}T.m/A)$, ${\chi }_{0}$ is the equilibrium susceptibility, H_0_ is the magnetic field amplitude (A/m), $f$ is the magnetic field frequency (Hz) and $\tau$ is the effective time constant that is determined using Eq. (4)^61^:

4 $${\tau }^{-1}= {\tau }_{N}^{-1}+ {\tau }_{B}^{-1}.$$

${\tau }_{N}$ is the Neel relaxation time and ${\tau }_{B}$ is the Brownian relaxation time. Neel relaxation is attributed to alignment of the magnetic moment of the nanoparticle with the magnetic field without rotating the nanoparticle itself that results in heat production in the nanoparticle and so nanofluids^61^, on the other hand at the Brownian relaxation the nanoparticle rotates with the magnetic field and produces heat due to friction between nanoparticle and the fluid^61^. ${\tau }_{N}$ and ${\tau }_{B}$ are determined by Eqs. (5) and (6)^5^:

5 $${\tau }_{N}= {\tau }_{0}{\text{exp}}\left(\frac{KV}{kT}\right),$$

6 $${\tau }_{B}=\frac{4\pi \eta {r}_{h}^{3}}{kT}.$$

In the above equations ${\tau }_{0}$ is the pre-exponential time constant, and variable amounts has been reported for it in literature between 10^–7^ and 10^–13^, in this research 3 × 10^–9^ will be used according to Berkov et al.^64^. K is the volume anisotropy constant (J.m^–3^) and for bulk magnetite is approximately 10^4^ J.m^-3^^5,58,65,66^ while the measurements showed that due to broken symmetry at the surface of the nanoparticles this parameter can increase up to 2.5 × 10^4^ J.m^–3^ in nanoparticles^67^. V is the volume of the magnetic core (m^3^), k is the Boltzmann’s constant (1.3806503 × 10^–23^ m^2^ kg s^–2^ K^–1^), T is the temperature, $\eta$ is the viscosity of the ferrofluids and r_h_ is the hydrodynamic radius of the nanoparticles. According to Eq. (4) in case of existing one or more orders of magnitudes difference between ${\tau }_{N}$ and ${\tau }_{B}$ the lower one will be a good estimate of the τ. As stated earlier ${\chi }_{0}$ is the equilibrium susceptibility and is determined using Langevin equation^46^ as follow;

7 $${\chi }_{0}= {\chi }_{i}\frac{3}{\xi }\left({\text{coth}}\xi -\frac{1}{\xi }\right).$$

At the Eq. (7) ${\chi }_{i}$ is the initial susceptibility and determined using the Eq. (8);

8 $${\chi }_{i}= \frac{{\mu }_{0}\phi {M}_{d}^{2}{V}_{m}}{3kT}.$$

$\xi$ is defined as $\xi = {\mu }_{0}{M}_{d}HV/kT$, ${M}_{d}$ is the domain magnetization of a suspended particle and defined as ${M}_{d}= {M}_{s}/\phi$ and finally $\phi$ is the volume fraction solids^46^.

The use of equilibrium susceptibility in cases where the field is alternating is somewhat questionable, and Rosensweig has not specified exactly whether the Langevin parameter should be determined using the peak amplitude of the alternating field or another value should be considered. But in research related to this matter, peak amplitude has been used and accurate answers have been obtained^68^. In order to determine the theoretical SAR values at first the Neel relaxation time and Brownian relaxation time must be measured and in order to determine the Brownian relaxation time constant of the nanoparticles in ferrofluid, the viscosity of polymer solution must be measured. The viscosity of the polymer solutions containing the nanoparticles was measured using QC viscometer (Anton Paar Company) as our previous work^56^. The polymer is shear thinning^56^ and as the solutions were stationary in heating test so the viscosity was determined at very low shear rate (0.1 1/s) to accurately estimate the viscosity of the stationary solution. Furthermore as the solution temperature rises during the heating tests, the viscosity was determined at different temperature between 20 °C and 90 °C.

### Experimental SAR measurement and final temperature

An AC magnetic field producer (LABA, iHT-1000W, NATSYCO) was used to conduct the heating tests. The 1 wt% polymer solution was prepared in order to obtain a viscous solution. Nanoparticles test concentrations was 20,000 ppm and 10,000 ppm, test frequency was about 100 kHz and magnetic field intensities was 8 kA/m, 10kA/m and 12 kA/m. The temperature was measured with an alcohol thermometer with an accuracy of 0.1 °C. The experimental specific absorption rate (SAR) values were determined using Eq. (9) as follow^3^:

9 $${\text{SAR}}=\left(\frac{1}{{{\text{w}}}_{{{\text{Fe}}}_{3}{{\text{O}}}_{4}}}\right){{\text{C}}}_{{\text{p}}}\left[\frac{\Delta {\text{T}}}{\Delta {\text{t}}}\right].$$

**Figure 2:**
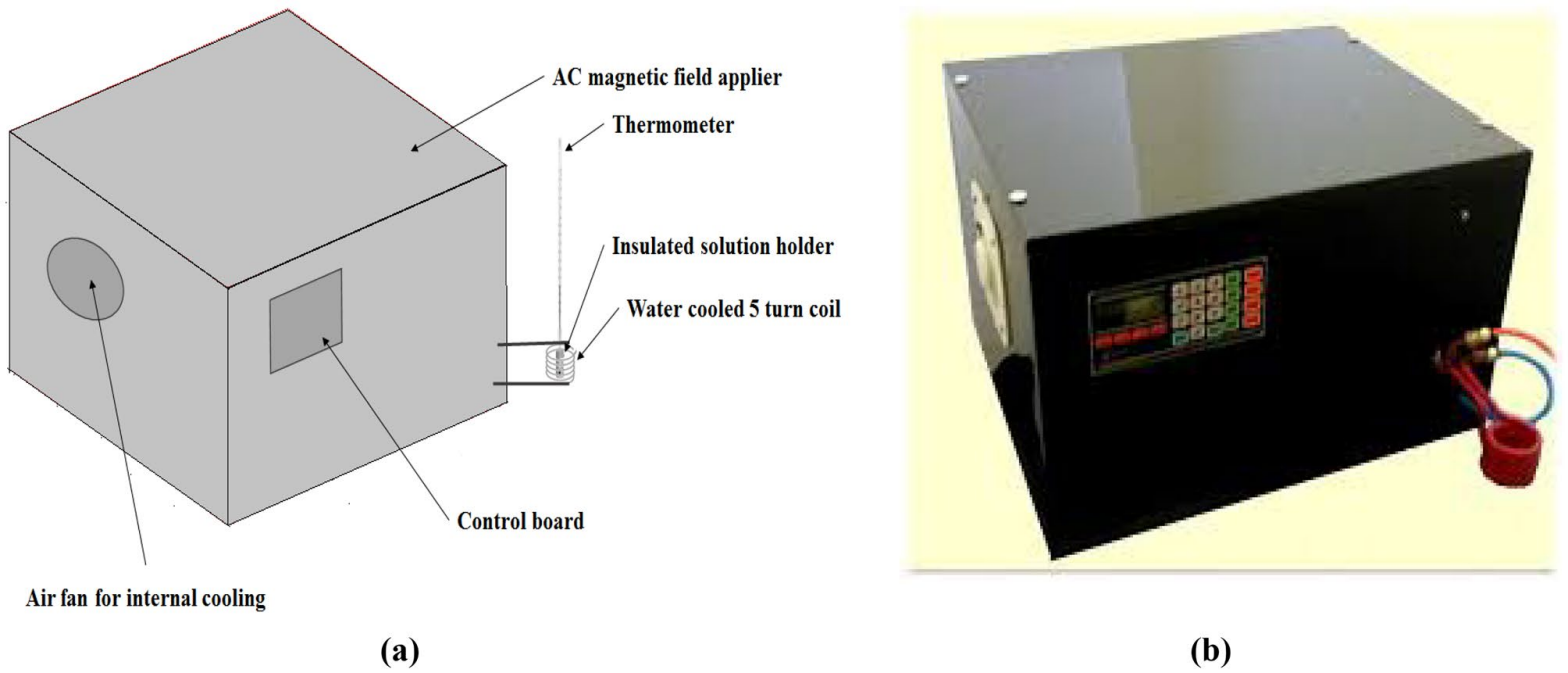
(**a**) Schematic representation of heating test setup (**b**) The AC magnetic field heating apparatus.

The amount of $\frac{\Delta T}{\Delta t}$ was determined at the beginning of the test when this variable was maximum, and the heat flow to environment was at the lowest possible amount. ${w}_{{Fe}_{3}{O}_{4}}$ is the weight fraction of nanoparticles in polymer solution. The specific heat capacity of dispersed nanoparticles in polymer solution was assumed to be equal to water specific heat capacity because of low concentration of the polymer and nanoparticles. The heating tests were continued until no temperature change occurred in the solutions and final temperature of the solutions were determined. The effect of surface modification, magnetic field intensity and nanoparticles concentration on the SAR and final temperature was investigated. A schematic drawing of the heating test set up and a picture of the AC magnetic field device is showed in Fig. 2.

## Results and discussion

### Nanoparticles’ characterization

**Figure 3:**
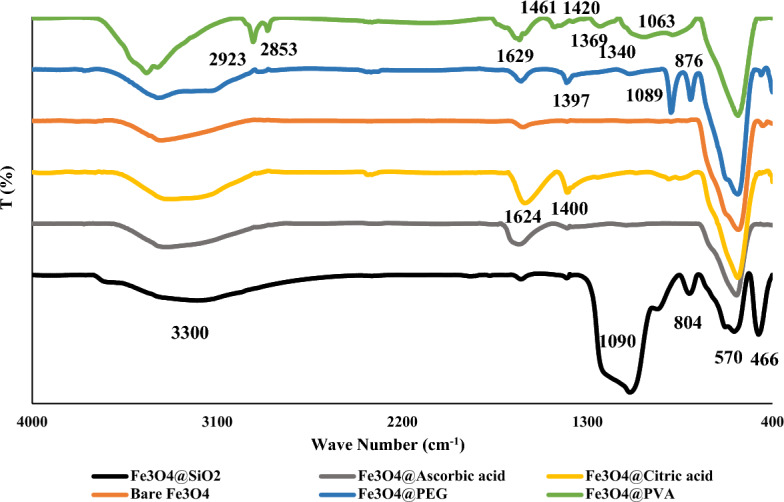
FTIR spectra of the synthesized nanoparticles.

The FTIR test results are shown in Fig. 3, and the peaks were characterized in Table 1. The common peaks in all spectrums (570 cm^–1^ and 3300 cm^–1^) belongs to Fe–O (Fe_3_O_4_) and O–H (H_2_O). H_2_O not only absorbs to the surface of the Fe_3_O_4_ molecule, but also all the surface modification agents that has been used in this project, except SiO_2_, have O–H in their structure and so this peak belongs to both surface absorbed H_2_O and the O–H in the formula. The FTIR spectra of carbonyl group in pure citric acid and ascorbic acid has absorption bond in wave number of about 1700 cm^–1^ but interaction with Fe–O bonding moves it to around 1600 cm^–1^^69^. The absorption wave number around 1080 for both Fe_3_O_4_ @PVA and Fe_3_O_4_@PEG is indicative of interaction between Fe atoms in Fe_3_O_4_ and C–O group of PVA and PEG (Supplementary Fig. 3).

**Table 1:** FTIR peaks of the nanoparticles.

Wave number (cm^–1^)	Characterization	Wave Number (cm^–1^)	Characterization
Common peaks		Fe_3_O_4_ @ PVA & Fe_3_O_4_ & PEG	
570	Fe–O^23,24,70^	876	CH_2_ rocking^71,72^
3300	H–O^69,73,74^	1063, 1089	Fe–O–C^71,72,75,76^
Fe_3_O_4_ @ SiO_2_		1340	–C–O asymmetric stretching^77^
466	Fe–O under the influence of Si–O–Si^73^	1369 (Fe_3_O_4_@PVA), 1397(Fe_3_O_4_@PEG)	CH_2_ twisting^71,77^
804	Si–O–Si^70^	1420	C–C ^72^
1090	Si–O^24^	1461	CH bending^71^
Fe_3_O_4_ @ Citric acid & Fe_3_O_4_ @ Ascorbic acid		1629	O–H bending^71,72^
1624, 1600	C=O at COO group^69,74^	2853	C–H stretching in CH_3_^71^
1400	C–O^69^	2923	C–H stretching in CH_2_^71,72,76,78^
2900	C–H^69^		

**Figure 4:**
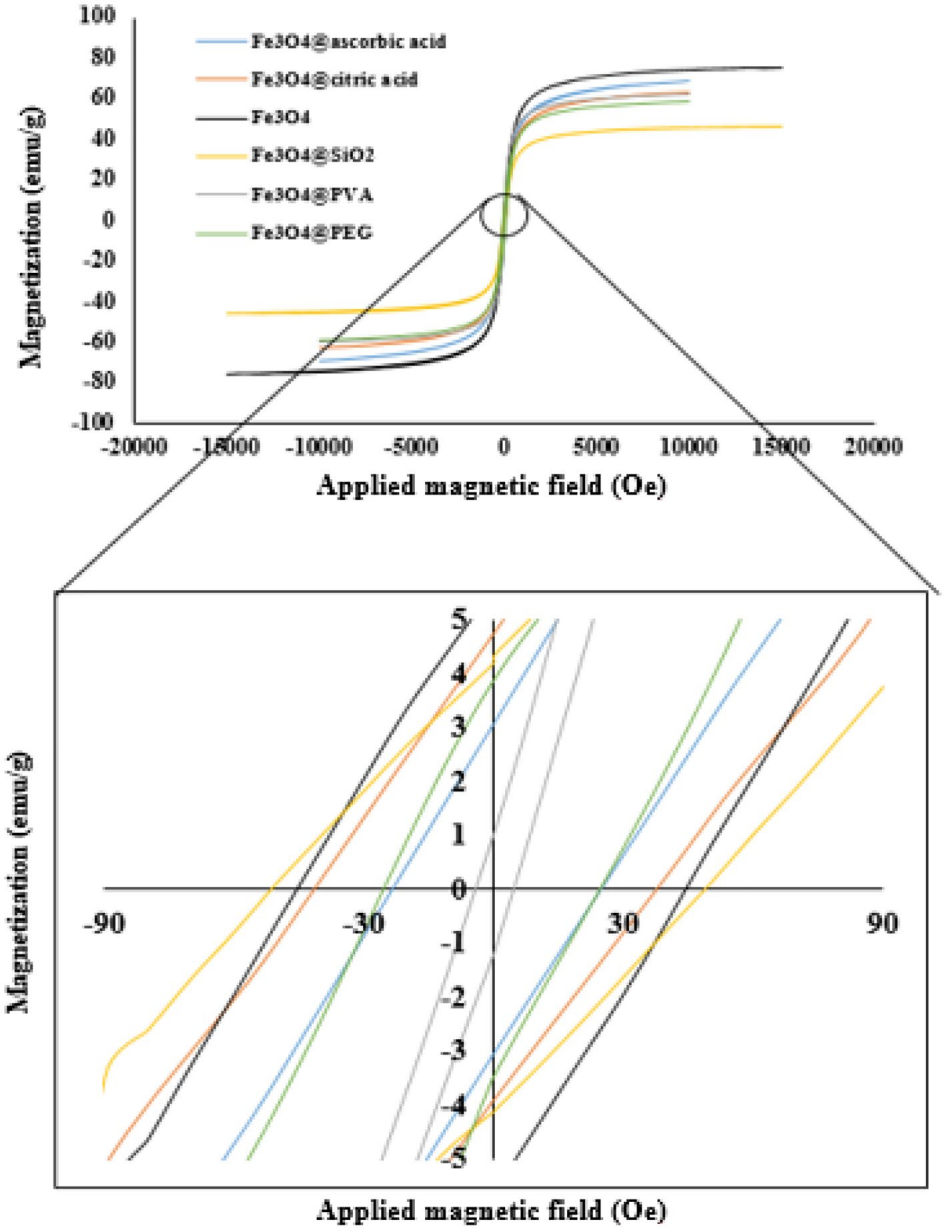
M-H curve for synthesized nanoparticles.

The results of VSM test is shown in Fig. 4. It is obvious that all synthesized nanoparticles are not ideal superparamagnetic nanoparticles but the coercivity is low enough to expect Neel relaxation mechanism from these nanoparticles. The coercivity and saturation magnetization and the magnetic core radius of the nanoparticles using the VSM tests results and Eq. (2) are tabulated in Table 2 (Supplementary Fig. 4). It is worth mentioning that an ideal superparamagnetic nanoparticle should have zero coercivity^79^, but on the other hand achivieng to exactly zero coercivity is not common in literature and different low amounts has been reported in literature for this parameter. For example 189.3 Oe^80^, 7.8 Oe^81^ and 41.7 Oe^82^. Comparing the results with literature shows that the results are in acceptable range and the hystersis loops are negligable^79,83–85^. However, the D_m_ obtained from VSM results is for the magnetic core of the nanoparticles and because of surface modifications, existence of some magnetically dead layer on the surface of the magnetic core is unavoidable and so the hydrodynamic radius of the nanoparticles were greater than r_m_ amounts^86,87^. It is also noteworthy that the magnetic core radius of the nanoparticles is at the same range and there is a little difference between the magnetic core radiuses of the nanoparticles that was because of robust control on the temperature, composition of the reagents and addition rate of the NH_4_OH to the reaction medium at synthesizing procedure.

**Table 2:** Saturation magnetization, coercivity and D_m_ of the nanoparticles.

Nanoparticle	Coercivity (Oe)	M_s_ (emu/g)	D_m_
Fe_3_O_4_	43	74.9	8.28
Fe_3_O_4_@PVA	5	62.7	8.32
Fe_3_O_4_ @ citric acid	38	63.7	7.80
Fe_3_O_4_ @ ascorbic acid	26	69.1	8.24
Fe_3_O_4_@PEG	27	58.9	8.92
Fe_3_O_4_ @ SiO_2_	48	45.6	8.77

It should be noticed that the coercivity and size of the hysteresis loop have decreased as temperature have increased and frequency have decreased^88–90^, as a result, it is reasonable to assume that the coercivity value will drop during the heating experiments in the magnetic field comparing to the value observed in the VSM test. The presence of nanoparticles in the dry state might cause interactions and the creation of larger–diameter structures, resulting in hysteresis loop^91^.

**Figure 5:**
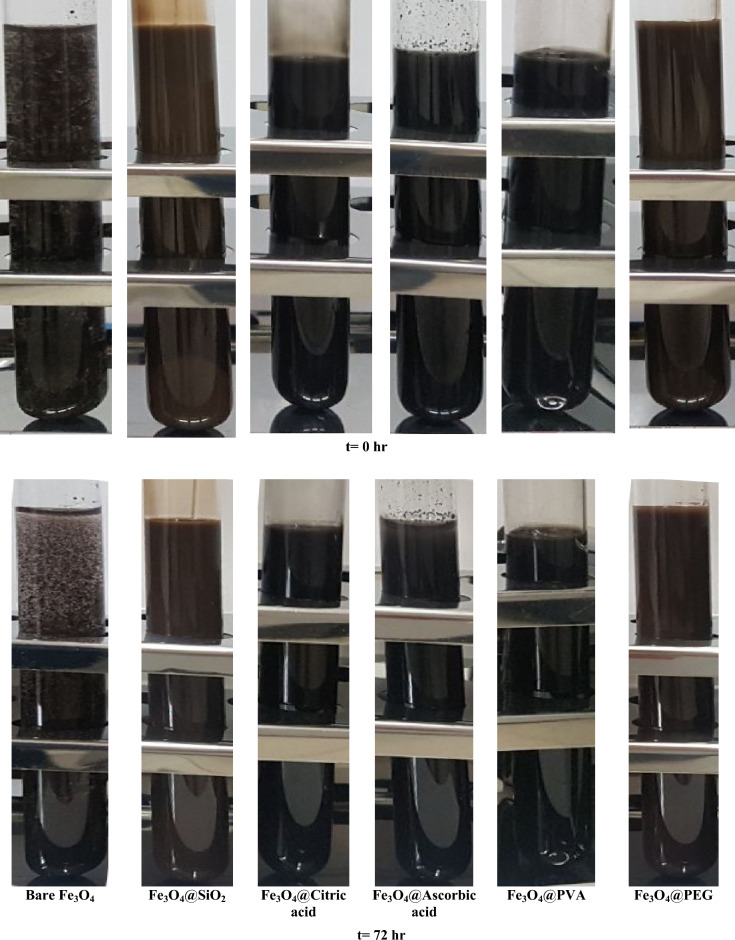
Stability of nanoparticles dispersion in polymeric solution, polymer concentration: 10,000 ppm, nanoparticle concentration: 20,000 ppm.

The effect of surface modification on stability of the dispersions was shown in Fig. 5. As it is obvious all the surface modified samples showed no perception after 72 h except dispersion containing bare Fe_3_O_4_ nanoparticles.

### Theoretical SAR values

**Figure 6:**
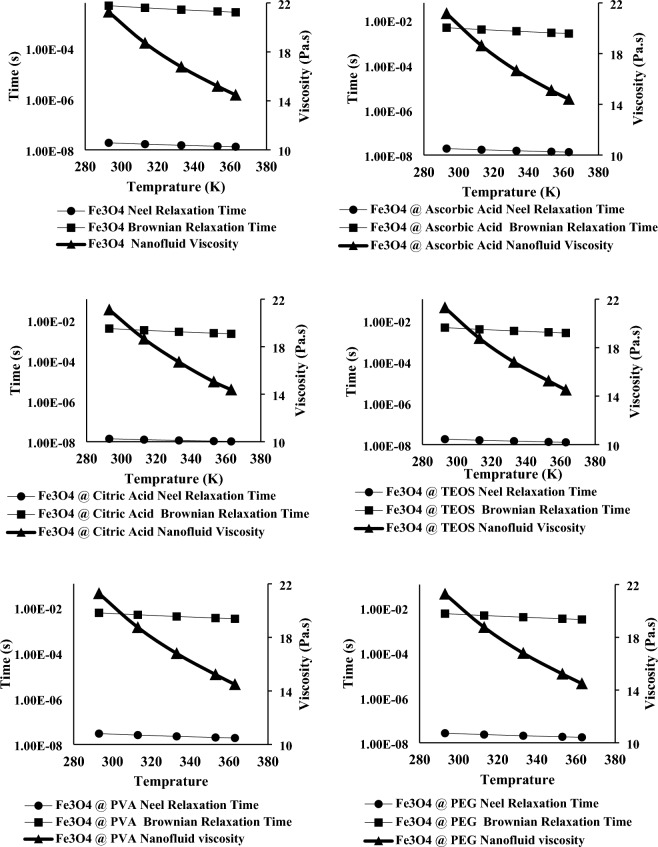
Nanoparticles Neel and Brownian relaxation times and viscosity of the nanofluids.

The viscosity of the nanofluids containing different surface modified Fe_3_O_4_ nanoparticles at different temperatures are presented in Fig. 6. As it is obvious the viscosity has decreased by increasing the temperature. The viscosity of the nanofluids are approximately equal that was predictable because the concentration of the nanoparticles was low and the polymer itself was the dominant factor in the viscosity of the nanofluids (Supplementary Fig. 6).

The Neel relaxation time and Brownian relaxation time constants at different temperatures for nanofluids containing synthesized nanoparticles using Eqs. (5) and (6) are also shown in Fig. 6. As it is obvious the Brownian relaxation time is much greater than Neel relaxation time in all dispersions ($\frac{{\tau }_{B}}{{\tau }_{N}} > {10}^{5}$) that was predictable, because the viscosity of the nanofluids was much greater than the nanofluid systems that water or solvents with viscosity near to water was used as dispersant. Also as mentioned earlier due to surface modification of the nanoparticles and existence of magnetically dead layer on the surface of the nanoparticles the r_h_ > r_m_, and as r_m_ was used for Brownian relaxation time measurement in this manuscript, so the real Brownian time constant was greater than the measured amounts. So, it can be said with high certainty that the effective time constant is equal to Neel relaxation time constant and this parameter was used in theoretical SAR values determination.

Of course, the important note that should be considered in this part is to ensure that the nanoparticles are in the linear response regime in order to be able to use Eq. (3). Linear response regime refers to a region where magnetization is linearly related to the magnetic field. Different criteria has been mentioned in order to evaluate this, first, the $\xi$ parameter must be smaller than 1^92,93^. The exact solution of the Shlimois relaxation equation has shown that the SAR value for $\xi$ parameter less than one equal to the SAR value obtained from the Rosensweig model^68^. For all the synthesized nanoparticles in this research, the value of the $\xi$ parameter was less than 0.7. Another criteria to ensure being within this region is that the range of applied field intensity must has a certain distance from the saturation intensity^94^. By reviewing the data obtained from the VSM test, it can be seen that in all samples up to 300 Oe (23.9 kA/m), a linear relation is obvious between magnetization and the intensity of the applied field, and up to this applied field, the amount of magnetization has a suitable distance from the saturation magnetization. On the other hand, the maximum applied magnetic field in the conducted experiments was equal to 12 kA/m, and therefore, considering both the first criteria and the second criteria, it can be concluded that the tests are done in the linear response regime. Furthermore it has been determined in literature that the tests performed at a field intensity of 15 kA/m and a frequency of 300 kHz on iron oxide-based nanoparticles are in the linear response region^93^. Due to the fact that in this research, the field intensity and applied frequency values have a significant distance from these values, therefore, it can be ensured that the experiments are carried out in the linear response region.

In addition to the $\xi$ parameter, which can be used as a criterion for formula selection, it has been demonstrated that when $\tau$ was less than 1/f (as it was in this study), the hysteresis loop and its area were very small^52^. At coercivity of 125 Oe and remanent magnetization of 16 emu/gr, the quantity of heat resulting from the hysteresis loops is approximately 50% of the total heat resulting from the heating operation in the setup, with relaxation accounting for the remainder^95^, As a result, because the quantity of coercivity and remanent magnetization, and thus the size of the hysteresis loop were lower in this study, it can be assumed that the amount of heat created by hysteresis is significantly lower, and the majority of the heat produced is due to relaxation.

Although there is no doubt that the simplification and omission of heat created by the hysteresis loop has introduced some mistakes in the computations. In other words, the numbers provided in Table 3 may be slightly higher than the values already listed, and the results produced from theoretical and experimental measurements are further apart. However, based on the explanations provided, it can be concluded that this simplification did not bring significant mistake into the calculations, and, based on the proper particle distribution, enough system insulation, and the similarity of the results of Table 4 to Table 3, it can be estimated that the neglected value in the theoretical calculations due to this simplification was just a small fraction.

**Table 3:** Theoretical SAR values for nanofluids.

Type of nanoparticle in nanofluid	Magnetic field amplitude (kA.m^–1^)	SAR (W/g)	Magnetic field amplitude (kA.m^–1^)	SAR (W/g)	Magnetic field amplitude (kA.m^–1^)	SAR (W/g)
Fe_3_O_4_	8	5.17	10	8.0	12	11.5
Fe_3_O_4_ @ citric acid	8	3.7	10	5.7	12	8.2
Fe_3_O_4_ @ ascorbic acid	8	4.8	10	7.5	12	10.7
Fe_3_O_4_ @ SiO_2_	8	3.1	10	4.8	12	6.9
Fe_3_O_4_ @ PVA	8	5.4	10	8.3	12	11.9
Fe_3_O_4_ @ PEG	8	4.8	10	7.5	12	10.7

**Table 4:** SAR (W/g) and final temperature (°C) at different magnetic fields for aqueous solutions containing 10,000 ppm of polymer and 20,000 ppm of nanoparticles.

Nanoparticle	Bare Fe_3_O_4_	Fe_3_O_4_ @ PEG	Fe_3_O_4_ @ PVA	Fe_3_O_4_ @ SiO_2_	Fe_3_O_4_ @ ascorbic acid	Fe_3_O_4_ @ citric acid
H = 8 kA/m						
SAR (w/g)	4.9	3.85	4.2	2.80	4.2	3.15
Final temperature (°C)	56.6	42.9	48.9	36.4	50.9	39.3

Nanoparticle	Fe_3_O_4_	Fe_3_O_4_ @ PEG	Fe_3_O_4_ @ PVA	Fe_3_O_4_ @ SiO_2_	Fe_3_O_4_ @ ascorbic acid	Fe_3_O_4_ @ citric acid
H = 10 kA/m						
SAR (w/g)	7.0	5.25	5.6	4.20	6.07	4.55
Final temperature (°C)	83.9	65.9	78.1	53.5	80.6	58.0
H = 12 kA/m						
SAR (w/g)	9.72	6.83	7.09	5.51	8.66	6.56
Final temperature (°C)	89.2	55.0	80.8	37.2	83.8	44.8

Considering the frequency of the tests (100 kHz) and VSM test results, Eq. (3) was used to determine the theoretical SAR values for the nanofluids at different magnetic field amplitudes and T = 22 °C and the results are tabulated in Table 3.

As it is obvious from the results the SAR values increases by increasing the magnetic field strength. It should be noted that the theoretical amounts are watt per gram of magnetic core, so the oxidation of the magnetite surface and also the surface modification could result in this parameter decrease in real experiments that will be discussed in the next section. Another point that should be noted is that according to the Eq. (3) in an ideal suspension without interaction between nanoparticles and coagulation of them, the SAR amount is independent of the nanoparticle’s concentration.

### Magnetic heating results

**Figure 7:**
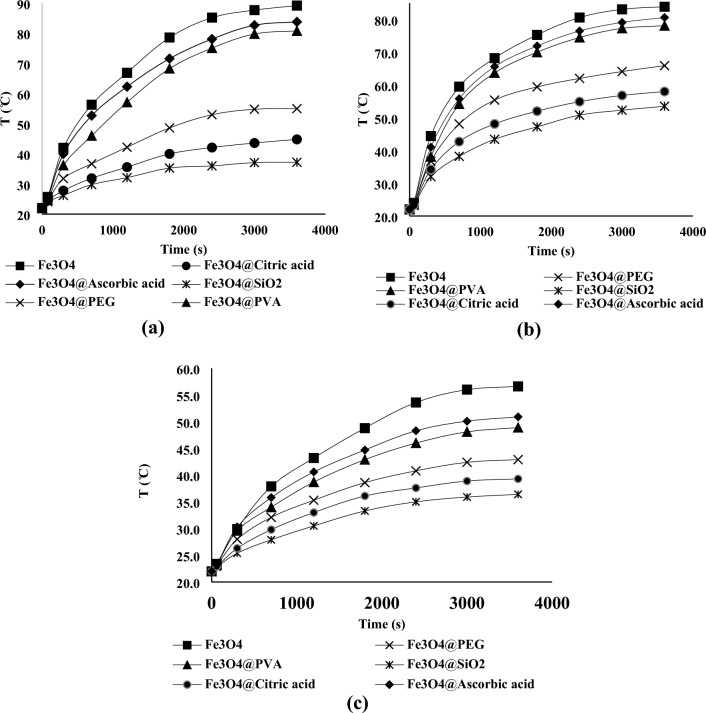
Temperature vs. time in (**a**) H = 12 kA.m^–1^ (**b**) H = 10 kA.m^–1^ (**c**) H = 8 kA.m^–1^ for solutions containing 20,000 ppm of nanoparticles.

The heating results in different magnetic fields for nanofluids containing 20,000 ppm of the nanofluids are shown in Fig. 7 (Supplementary Fig. 7).

**Figure 8:**
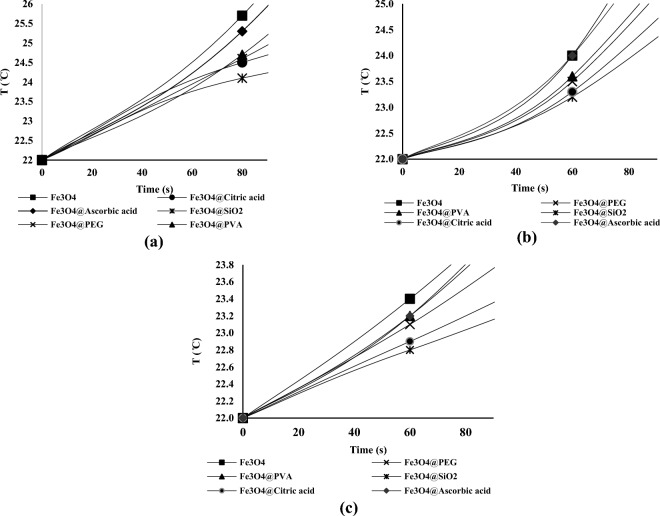
Temperature vs. time in (**a**) H = 12 kA.m^–1^ (**b**) H = 10 kA.m^–1^ (**c**) H = 8 kA.m^–1^ for solutions containing 20,000 ppm of nanoparticles at the beginning of the test.

The SAR values using Eq. (9) are given in Table 4. It is worth mentioning due to low concentration of polymer and nanoparticles C_p_ of the solution was considered equal to C_p_ of water. It is also worth mentioning that ($\frac{dT}{dt}$) in the first 60 or 80 s are used in calculating theoretical SAR. The heating test results at the beginning of the test (first 90 s of the Fig. 7 diagrams) are tabulated in Fig. 8.

The non-linearity of the graphs even in the first 60 or 80 s is visible. It is clear that assuming this petameter to be linear in the calculations of the produced heat causes errors, and it appears that shortening the time period for measuring the initial temperature changes can lead to more accurate results. However, it should be noted that shortening the measurement duration can result in errors for the following reasons:

- Even a small error in temperature measurement can lead to a large error in $\frac{dT}{dt}$ value. Because the denominator of this fraction also becomes very small.
- The temperature measuring device’s accuracy was just 0.1 °C, so it was impossible to discern between temperature variations brought on by various nanoparticles within a few seconds.
- Reduced measurement duration causes the tester’s inaccuracies during temperature measurement (in the range of a few seconds) to lead to a substantial inaccuracy in the value of $\frac{dT}{dt}$, therefore it is advisable to use a relatively greater time range.

Taking into account everything mentioned above, it was determined that the best time period for measuring initial temperature changes was the first 60 and 80 seconds of the tests.

Comparison between the theoretical SAR values at Table 3 and experimental results in Table 4 shows that the experimental amounts are lower. In Eq. (3), it is clear that the SAR is a 2nd order function of the H. The parameter that is defined according to this functionality in various researches is intrinsic loss power (ILP), which is defined as Eq. (10)^94^.

10 $$ILP = \frac{SAR}{{H}^{2}f}.$$

**Figure 9:**
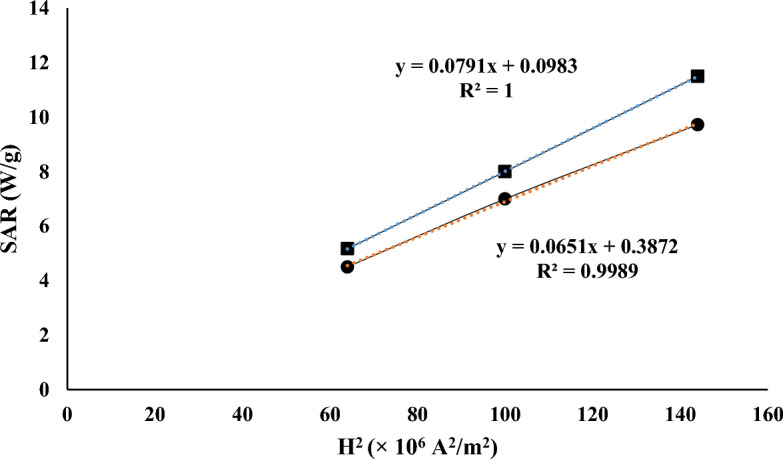
Theoretical and experimental heating results of bare Fe_3_O_4_.

ILP is a system-independent parameter that allows direct comparison of tests performed in different laboratories. If the frequency value is constant in a system, it can be concluded that the dependence of SAR on H^2^ should be linear. Therefore, this relationship was used to check the validity of experimental SAR results in terms of H^2^ and comparing it with theoretical results. Although this relationship has been shown to be true up to field strengths of about 20 kA/m^68^. In case of bare Fe_3_O_4_ the theoretical and experimental results per H^2^ are plotted in Fig. 9. The experimental results are a little (10–15%) less than theoretical expectations (in case of bare Fe_3_O_4_ nanoparticles). Although this amount of difference is an acceptable value for an experimental experiment, several reasons can be mentioned for this amount of difference. As mentioned in the introduction, many efforts have been made so far to formulate magnetic heating and the effect of various factors on the difference in the reports provided by various researches^93^. Considering these researches as well as the conditions of the experiment conducted in our research, the following factors can be mentioned as the reasons for the deviation in the results of the experiments compared to the theoretical results^46,47,68,92–94,96,97^:

1. Absence of complete adiabatic conditions in the system.
2. Non-establishment of the conditions related to the linear response region due to the high intensity of the applied magnetic field.
3. The produced nanoparticles had a hysteresis loop and were not ideal superparamagnetic nanoparticles.
4. The low accuracy of the measuring instrument in determining $\frac{dT}{dt}$ at the initial moment for various reasons such as inaccuracy in temperature measurement, uncertainty in placing the probe, etc.
5. The occurrence of aggregation, agglomeration clustering, sedimentation and to a small extent chemical reaction.
6. Uncertainty in the measurement of magnetic field intensity.
7. The possibility of the effect of sample aging on laboratory results.
8. Non-uniformity of applied magnetic field.
9. Rosensweig’s model for calculating SAR gives an upper limit of SAR and there is a possibility that the actual SAR is slightly lower.

Pearson correlation coefficient was used to check the compatibility between experimental and theoretical data^97^. The value of this parameter between these two datasets is equal to 0.999. This coefficient is a measure of the linear relationship between these datasets.

An approximately linear relation (with y-intercept near to zero) between SAR and H^2^ is obvious in the Fig. 9 in both theoretical and experimental results and this relation is clear in Eq. (3), also ${\chi }_{0}$ is dependent on applied magnetic field and so there is a little deviation from exact linear relation. The difference between theoretical and experimental SAR amounts are less in lower magnetic field strengths that is because of the lower temperature of the dispersion in this case that is an evidence for thermal losses of the system.

In case of surface modified nanoparticles the difference between theoretical and experimental SAR amounts are larger. The reason is that the theoretical SAR amounts are based on weight of magnetic portion of the nanoparticle but in experimental tests the SAR amounts are based on nanoparticles weight (magnetic core and surface modification agent). As previously mentioned in material and method section the amount of used surface modification agents were 40 wt% of the nanoparticles, also not all of the surface modification agent adhere to the surface of the nanoparticle and part of them that has been attached physically -not chemically- on the surface of the magnetite has been eliminated during the washing of the nanoparticles. Figure 9 can also be used to investigate the effect of hysteresis loops on the heating process. Since linear behavior is observed in the quantity of heat absorbed in terms of H^2^ (which is a representation of ILP), the heat produced by nanoparticles in the heating measurement tests in the alternating magnetic field was in the linear response regime and so the particles acted like nanoparticles with no coercivity^98^.

The effect of the surface modification on the experimental SAR of nanoparticles can also be investigated considering the changes in the amount of saturation magnetization. The decrease in the saturation magnetization of the surface modified nanoparticles compared to the bare nanoparticle is caused by the surface modification agent, which is a non-magnetic material. The ratio of saturation magnetization of surface modified nanoparticles to the saturation magnetization of bare nanoparticles, as well as the ratio of experimental SAR value of surface modified nanoparticles to the experimental SAR values of bare nanoparticles are given in the Table 5.

**Table 5:** The effect of Ms decrease on SAR.

Type of nanoparticle in nanofluid	M_s_/M_s,Bare nanoparticle_	SAR/SAR_Bare nanoparticle_		
		H = 8 kA.m^–1^	H = 10 kA.m^–1^	H = 12 kA.m^–1^
Fe_3_O_4_ @ citric acid	0.85	0.64	0.65	0.67
Fe_3_O_4_ @ ascorbic acid	0.92	0.86	0.87	0.89
Fe_3_O_4_ @ SiO_2_	0.61	0.57	0.60	0.57
Fe_3_O_4_ @ PVA	0.84	0.86	0.80	0.73
Fe_3_O_4_ @ PEG	0.79	0.79	0.75	0.70

Except for citric acid, there is a good correlation between these two ratios in the rest of the nanoparticles. In other words, the effect of adding non-magnetic materials has appeared both in reducing saturation magnetization and in the amount of heat produced. In the case of citric acid, it should be noted that the size of nanoparticles modified with citric acid is smaller compared to other nanoparticles, which leads to a decrease in the theoretical SAR of this nanoparticle and ultimately leads to a decrease in its experimental SAR value^68^. The smaller size of citric acid coated nanoparticle is due to the very good surface modification of Fe_3_O_4_ nanoparticle by this agent, which prevents any coagulation of it, and it has also been discussed in our previous paper^56^. The ratio of experimental to theoretical SAR values at different magnetic field are brought in Table 6. The trend of decreasing the ratio by increasing the magnetic field is obvious.

**Table 6:** The ratio of experimental SAR to theoretical SAR for test samples, C_nanoparticle_ = 20,000 ppm.

Magnetic field (kA/m)	Fe_3_O_4_	Fe_3_O_4_ @ SiO_2_	Fe_3_O_4_ @ascorbic acid	Fe_3_O_4_ @ citric acid	Fe_3_O_4_ @ PVA	Fe_3_O_4_ @ PEG
8	0.95	0.90	0.87	0.85	0.78	0.80
10	0.87	0.87	0.81	0.80	0.67	0.70
12	0.85	0.80	0.81	0.80	0.60	0.64

Another issue that should be pointed out is that the $\frac{{SAR}_{Experimental}}{{SAR}_{Theoretical}}$ is lower in case that PVA and PEG was used for surface modification of the nanoparticles. This phenomena is due to the larger molecules of polymers comparing the molecules of SiO_2_, citric acid and ascorbic acid. In other words the $\frac{{m}_{magnetitic core} }{{m}_{nanoparticle}}$ in Fe_3_O_4_ @ PVA and Fe_3_O_4_ @ PEG was lower comparing the other three surface modification agents.

**Figure 10:**
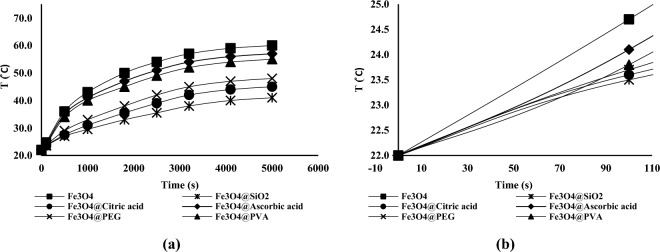
Temperature vs. time in H = 12 kA.m^–1^ for solutions containing 10,000 ppm of nanoparticles (**a**) in 5500 s (**b**) in 110 s.

The heating test results for dispersions containing 10,000 ppm of nanoparticles at magnetic field of 12 kA.m^–1^ is shown in Fig. 10 and experimental SAR values and final temperatures are tabulated in Table 7 (Supplementary Fig. 10).

**Table 7:** SAR values (W/g) and final temperature (°C) for dispersions containing 10,000 ppm of nanoparticles, H = 12 kA.m^–1^.

Nanoparticle	Fe_3_O_4_	Fe_3_O_4_ @ PEG	Fe_3_O_4_ @ PVA	Fe_3_O_4_ @ SiO_2_	Fe_3_O_4_ @ ascorbic acid	Fe_3_O_4_ @ citric acid
SAR (w/g)	11.34	7.14	7.56	6.30	8.82	6.72
Final temperature (°C)	60.0	48.0	55.0	41.0	57.0	45.0

It is clear that experimental SAR values in case of 10,000 ppm concentration of nanoparticles was more than 20,000 ppm samples. This phenomena has been observed in similar researches, too^4^. In case of solutions with higher concentrations the coagulation possibility would increase and so the amount of experimental SAR amount would decrease by increasing the nanoparticles concentrations. For investigating the effect of surface modification agent on coagulation and agglomeration inhibition of the system the ratio of experimental SAR in case of 20,000 ppm of nanoparticles to experimental SAR in case of 10,000 ppm of nanoparticles ($\frac{{SAR}_{20000 ppm}}{{SAR}_{10000 ppm}})$ in magnetic field intensity of 12 kA.m^–1^ are tabulated in Table 8.

**Table 8:** The experimental $\frac{{{\text{SAR}}}_{20000\mathrm{ ppm}}}{{{\text{SAR}}}_{10000\mathrm{ ppm}}}$ at H = 12 kA.m^–1^.

Fe_3_O_4_	Fe_3_O_4_ @ PEG	Fe_3_O_4_ @ PVA	Fe_3_O_4_ @ SiO_2_	Fe_3_O_4_ @ ascorbic acid	Fe_3_O_4_ @ citric acid
0.86	0.96	0.94	0.87	0.98	0.98

It is obvious that the experimental SAR amounts decreases more by increasing the nanoparticle concentration in nanofluid samples containing Fe_3_O_4_ and Fe_3_O_4_@SiO_2_. These two nanoparticles have lower hydrophilic behavior comparing other surface modified nanoparticles and so tends to agglomerate and form bigger clusters.

## Conclusion

The Neel mechanism on the heating of the nanofluids was investigated successfully and the experimental trends was as the theoretical trend especially in low concentrations (10,000 ppm). Surface modification right after the synthesis of the nanoparticles resulted in agglomeration prevention of the nanoparticles and so the magnetic core radius of the bare nanoparticles and all surface modified nanoparticles were the same and were about approximately 8 nm. Surface modification of Fe_3_O_4_ nanoparticles cause in decreasing the induction heating ability of the nanoparticles per gram of the nanoparticles that was because of magnetically dead layer on the surface of the nanoparticles, in other words the amount of effective substance in induction heating have decreased in surface modified nanoparticles. The amount of decrease in experimental SAR comparing theoretical SAR was more in case that PVA and PEG was used as surface modification agent that was because of bigger molecules of polymers comparing other surface modification agents. The experimental SAR to theoretical SAR was lower in higher magnetic field strengths that was because of thermal losses of the system that was more in higher temperatures. According to the theoretical relation the SAR amount is independent of nanoparticles concentration but in experimental results the SAR decreased by increasing the nanoparticles concentration that was because of agglomeration of nanoparticles. Surface modification prevents the nanoparticles from agglomeration, so the SAR in surface modified nanoparticles is less dependent on the nanoparticles concentration but in case of bare Fe_3_O_4_ and Fe_3_O_4_@SiO_2_ the hydrophobicity is low and so agglomeration of nanoparticles resulted in SAR decrease.

When there is a substantial coercivity value and the ξ parameter is greater than 1, the hysteresis loop should also be taken into account in the generated heat computations. In other words, the heat produced by nanoparticles is exactly equal to the heat produced by relaxation only when the particles are ideal superparamagnetic nanoparticles with zero coercivity. However, when this parameter is not zero and a hysteresis loop occurs in VSM tests, some heat (however minor) is created due to hysteresis, of course, depending on the frequency and quantity of saturation magnetization and coercivity (in general, the area of the hysteresis loop), this heat can be substantial. Of course, in cases where the heat generated by the hysteresis loop is taken into account in the calculations, it should not be limited to the measurement of the static hysteresis loop, but also the measurement of the dynamic hysteresis loop as well as the hysteresis loop in real test conditions (dispersion of the nanoparticles in test medium) and its changes during the test (by changing temperature and frequency) must be considered in the calculations.

### Supplementary Information

Supplementary Figure 3.

Supplementary Figure 4.

Supplementary Figure 6.

Supplementary Figure 7.

Supplementary Figure 10.

## Data Availability

All data generated or analyzed during this study are included in this published article [and its supplementary information files].
